# Research on Learning Evaluation of College Students Based on AHP and Fuzzy Comprehensive Evaluation

**DOI:** 10.1155/2022/9160695

**Published:** 2022-11-08

**Authors:** Henan Wu, Xiaorui Sun, Dejun Li, Yan Zhao

**Affiliations:** ^1^Aviation University of Air Force, Changchun 130022, China; ^2^School of Chemistry and Environmental Engineering, Changchun University of Science and Technology, Changchun 130022, China

## Abstract

The aim of this paper is to propose a scientific and practical evaluation method for college students' learning evaluation. The students' learning experience and harvest are the ultimate embodiment of colleges teaching quality. Considering the multilevel and fuzzy characteristics of learning evaluation, a novel college evaluation method is put forward based on the analytic hierarchy process and fuzzy comprehensive evaluation. The flow chart and index system for college students' learning evaluation are designed. Meanwhile, the weight of the evaluation index and the calculation steps of fuzzy evaluation is demonstrated and applied actually. The results show that the method is practical which has advantages over the simple analytic hierarchy process.

## 1. Introduction

In the west, as early as the 1980s, the famous scholar pace and Boyer proposed that the quality of students in the whole learning process should be paid attention to and taken as the core content of teaching quality monitoring in colleges and universities [1]. The learning centered evaluation has attracted more and more attention in international higher education evaluation. The students' learning experience and harvest have become the focus which drive the transformation of higher education evaluation from the final emphasis on “teaching” to “learning” [2]. In the past 20 years, the concept of student-centered and learning-centered evaluation has continuously led to the transformation of higher education teaching quality evaluation to the learning paradigm and has gradually become an important research field of learning evaluation for college students in China [3]. To promote the connotative development of higher education, the core is quality, and the key is evaluation. [4] Therefore, the evaluation of college Students' learning is not only the premise of improving teaching and talent training methods, but also the basic work to promote the connotative development of China's higher education.

Chinese educators have made great progress in college students' learning evaluation after years of research and unremitting efforts, such as broadening the research dimension in evaluation content, combination of technology and humanities in evaluation method, unity of instrumental, and value rationality in the evaluation index, which involved foreign excellent research results [3]. However, there are still some deficiencies in China's college students' learning evaluation. For example, in terms of evaluation indicators, there is a phenomenon of emphasizing indicators rather than weights, considering that there are defects in the subjectivity and randomness of weight distribution. Nevertheless, the establishment of an index system and the determination of weight are exactly the key and basis for the implementation of college students' learning evaluation [5]. Therefore, improving ideas should be proposed.

## 2. Relevant Research Methods

At present, there are many methods about students' learning evaluation.

Wan used the fuzzy comprehensive analysis method to evaluate the effect of middle school English classroom teaching. The method is concluded that can more objectively reflect teachers' teaching ability through practice [6]. Cheng improved the analytic hierarchy process, it was applied to evaluate college students' comprehensive quality, and the method was verified rationality and effectiveness through experiments [7]. Chen constructed a class management evaluation model using mathematical modeling ideas and analytic hierarchy process, which is based on the sorting and statistics of the questionnaire [8]. Dong and Dai designed a comprehensive evaluation system for the learning effect of college mathematics courses. They conducted an empirical study on the learning effect of college mathematics courses by using the analytic hierarchy process and fuzzy evaluation method [9]. Wang used fuzzy comprehensive evaluation to evaluate college student cadres, an evaluation system for college student cadres was established with quantitative scores [10]. Gao et al. determined the weight of learning ability evaluation indicators and constructed an evaluation system based on the factor load of the structural equation model, which with data collection and exploratory and confirmatory factor analysis [11].

From the above-given analysis, it can be concluded that the analytic hierarchy process and fuzzy comprehensive evaluation method are widely used in the education field, which can provide a simple, practical, and effective method for many schools' education decision-making. However, the analytic hierarchy process and fuzzy comprehensive evaluation method also have many shortcomings. For example, the analytic hierarchy process is selected from the original scheme, which cannot provide a new scheme for decision-making with less quantitative data and more qualitative components, and it is not easy to convince. The weight vector does not match the fuzzy matrix when using the fuzzy comprehensive evaluation method, which will cause the result to appear a superfuzzy phenomenon with poor resolution. It can even cause the evaluation failure.

**Figure 1:**
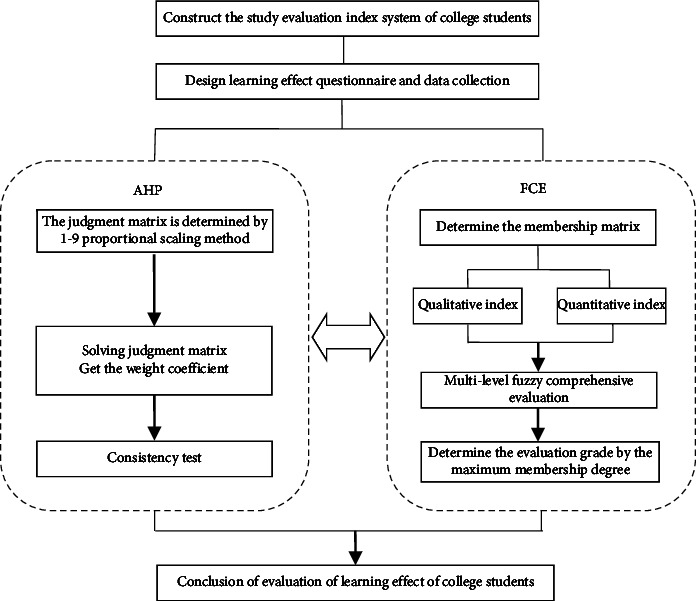
Flow chart of college students learning evaluation based on AHP-FCE.

The combination of the analytic hierarchy process and fuzzy comprehensive evaluation can overcome their own shortcomings and with greater advantages. When the analytic hierarchy process is adopted, the weight is more in accord with the objective reality and easy to express quantitatively, so as to improve the reliability, accuracy, and objectivity of fuzzy comprehensive evaluation; the fuzzy comprehensive evaluation method is reduced to the qualitative expression of the evaluation grade and each evaluation index, which makes the qualitative analysis and quantitative analysis better integrated. The paper constructs a new method with combining the advantages of the analytic hierarchy process and the fuzzy comprehensive evaluation method. The method was applied to evaluate College Students' learning, which not only comprehensively and systematically investigates the factors that affect college students' learning but also better resolves the fuzzy bottleneck in the evaluation process. The evaluation elements are accurately quantified. The calculation process refers to the corresponding scientific theories and measurement methods. It greatly improves the objectivity and accuracy of College Students' learning evaluation. The specific evaluation process is shown in Figure 1.

### 2.1. Analytic Hierarchy Process

**Figure 2:**
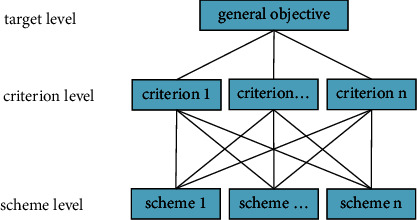
The model of analytic hierarchy process structure.

Analytic hierarchy process (AHP) is a hierarchical weight decision analysis method proposed by American operations research scientist professor T. L Saaty in the early 1970s. It has the characteristics of systematization, hierarchy, and multicriteria. AHP is often used in the selection of decision schemes for a complex problem with a wide range of applications. Its basic method comprises establishing a hierarchical structure model, and the steps are as follows: ① identify the problems that require decision-making and analyze the factors involved in the problems and their relationship. ② The decision-making problem is divided into different levels, namely, target level, criterion level, and scheme level in order. The model structure diagram is established and shown in Figure 2.

The mathematical process is illustrated by taking the hierarchical single sorting solution process as an example.

#### 2.1.1. Judgment Matrix

The judgment matrix is the core of the analytic hierarchy process.

(1) Concept: set *W*_*i*_ be the superiority of the scheme *i* for a target at the lowest level, and the matrix *A* with the weight number calculated by any two subgoals as the element, the judgment matrix.
  (1) $$\begin{array}{c} A=\left[\begin{array}{cccc} \frac{w_{1}}{w_{1}} & \frac{w_{1}}{w_{2}} & . & \frac{w_{1}}{w_{n}}\\ \frac{w_{2}}{w_{1}} & \frac{w_{2}}{w_{2}} & . & \frac{w_{2}}{w_{n}}\\ . & . & . & .\\ \frac{w_{n}}{w_{1}} & \frac{w_{n}}{w_{2}} & . & \frac{w_{n}}{w_{n}} \end{array}\right]\text{.} \end{array}$$
(2) Determination of each element in judgment matrix scale.

Take the judgment and quantification of the relative importance between any two factors as scale. The 1–9 scale methods are listed in Table 1.

Set *a*_*ij*_=*w*_*i*_/*w*_*j*_, the element *a*_*ij*_ of the judgment matrix has the following three properties: ① *a*_*ii*_=1;② *a*_*ij*_=1/*a*_*ji*_; ③ *a*_*ij*_=*a*_*ik*_*a*_*kj*_.

$A=\left[\begin{array}{cccc} a_{11} & a_{12} & . & a_{1n}\\ a_{21} & a_{22} & . & a_{2n}\\ . & . & . & .\\ a_{n1} & a_{n2} & . & a_{nn} \end{array}\right]$ The complete consistency judgment matrix satisfies the above three properties. The maximum characteristic root of the n-order complete consistency judgment matrix is *λ*_max_=*n*, and the other characteristic roots are 0.

#### 2.1.2. The Method to Determine the Weight

(1) Calculate the product *Mi* of each row element of the judgment matrix *P*
  (2) $$\begin{array}{c} M_{i}=\overset{N}{\underset{j=1}{\prod }}u_{ij},i=1,2,N\text{.} \end{array}$$
(2) Calculate the *n*-th root *Wi* of *Mi*
  (3) $$\begin{array}{c} \bar{W_{i}}=\sqrt[{n}]{M_{i}},i=1,2,\ldots ,N\text{.} \end{array}$$
(3) Normalize the vectors
  (4) $$\begin{array}{c} W_{i}=\frac{\bar{W_{i}}}{\sum_{j=1}^{n}\bar{W_{j}}}\text{.} \end{array}$$
*W*=[*W*_1_, *W*_2_, .,*W*_*n*_]^*T*^ is the feature vector, whose elements are weight coefficients
(4) Calculate the maximum eigenvalue of the judgment matrix (*PW*)_*i*_ represents the *i*-th element of vector *PW* [12]

#### 2.1.3. Consistency Test

Once the judgment matrix is constructed, it will be used to calculate the weights of any two elements in a criterion layer and verify the consistency after obtaining the results.

(1) The indicator *CI* measures the deviation of the judgment matrix from consistency. It is calculated by the following:
  (5) $$\begin{array}{c} CI=\frac{\lambda_{\mathrm{Max}}-n}{n-1}\text{.} \end{array}$$
Here, the value of *CI* is directly related to the consistency of the judgment matrix. The larger the *CI* value, the worse the consistency of the judgment matrix; when *CI* is 0, the judgment matrix has complete consistency.
(2) Mean random consistency index-RI. RI is the arithmetic average of the consistency index of *n* (*n*⟶*∞*) random judgments matrices. The values of the 3–9 order matrix RI are shown in Table 2.
(3) Inspection coefficient
  (6) $$\begin{array}{c} CR=\frac{CI}{RI}. \end{array}$$

The value of *CR* is directly related to the consistency of the CR ≤ 0.1, the judgment matrix has satisfactory consistency. Otherwise, the judgment matrix needs to be adjusted and then calculated until CI ≤ 0.1 to achieve satisfactory consistency.

#### 2.1.4. Hierarchical Total Ordering

Using the calculated single ranking results of the next layer in the process of calculating the weight of elements in this layer, which is the hierarchical total ranking. The order is conducted from low to high, and the total ranking of the highest layer is the single ranking result of its level. The consistency test formula of hierarchical total ordering is

(7) $$\begin{array}{c} CR=\frac{\sum_{i=1}^{n}w_{i}CI_{i}}{\sum_{i=1}^{n}W_{i}RI_{i}}\text{.} \end{array}$$

If the total sorting of all elements *A*_*1*_, *A*_*1*_,…, *A*_*m*_ in this level has been completed, the weights obtained are *a*_1_, *a*_2_,…, *a*_*m*_, the hierarchical single ordering result of all elements *B*_*1*_, *B*_*2*_,…, *B*_*n*_ corresponding to *a*_*k*_ at the upper level is: (*b*_1_^*k*^, *b*_2_^*k*^, .,*b*_*n*_^*k*^)′, when *B*_*i*_ is disconnected from *A*_*k*_, *b*_*i*_^*k*^=0 [7, 8].

### 2.2. Fuzzy Comprehensive Evaluation Method

Fuzzy comprehensive evaluation (FCE) is the method that judges matters based on fuzzy mathematical tools. It has some incomparable advantages over other comprehensive evaluation methods. Its operation principle is simple and easy to understand, and it is good at solving multi-factor and multilevel complex evaluation phenomena [7, 13].

The principle of FCE is to determine the evaluation index set *U*=(*U*_1_, *U*_2_, ., *U*_*n*_) and evaluation set *V*=(*V*_1_, *V*_2_, , *V*_*m*_) firstly, wherein *U*_*i*_ is the each single index and *V*_*j*_ is the evaluation level of *U*_*i*_, and then clarify the weight *W* of each variable and the belonging degree vector *R.* The fuzzy evaluation matrix *R* is obtained using the fuzzy method, and the matrix and weight vector are completed by fuzzy processing and normalization operation to get result *B.* The comprehensive evaluation model is formed and the specific steps are as follows.

#### 2.2.1. Determining the Evaluation Index Set *U* of the Evaluation Object

There are *n* evaluation indicators, *U*=(*U*_1_, *U*_2_, ., *U*_*n*_).

#### 2.2.2. Affirmation of the Evaluation Set V

*V*=(*V*_1_, *V*_2_, ., *V*_*m*_), each grade can be compared with the same fuzzy subset.

#### 2.2.3. Establishment of Fuzzy Relation Matrix *R* (Membership Matrix)

After the fuzzy subset construction is completed, all the evaluation factors are quantified to determine the membership of all fuzzy subsets. The fuzzy relation matrix formula is obtained as follows:

(8) $$\begin{array}{c} R=\left[\begin{array}{cccc} r_{11} & r_{12} & . & r_{1m}\\ r_{21} & r_{22} & . & r_{2m}\\ . & . & . & .\\ r_{n1} & r_{n2} & . & r_{nm} \end{array}\right]\text{.} \end{array}$$

The *r*_*ij*_ located in row I and column *J* represents the membership degree of fuzzy subsets of *U*_*i*_ to *V*_*j*_ in formula *R*, so *R* is also the membership matrix. The factor *U*_*j*_ indicates the existence of something which are obtained through *R*. Therefore, fuzzy comprehensive evaluation requires more information.

#### 2.2.4. Determining the Weight Vector W of Evaluation Factor

FCE method uses an analytic hierarchy process to determine the weight vector *W*=(*W*_1_, *W*_2_, ., *W*_*n*_) of evaluation factors.

This method is used to determine the relative importance of factor determination, clarify the weight coefficient and then make normalization processing.

#### 2.2.5. The Synthesis of Fuzzy Comprehensive Evaluation Result Matrix B

The most common method is the principle of maximum membership. The comment with the highest degree of membership is the evaluation result.

## 3. Construction of Learning Evaluation Model for College Students

### 3.1. The Framework of Learning Evaluation Model for College Students

It is a process of quantitative and qualitative evaluation of college education level and teaching effect to evaluate college students' learning. According to the research of Cai [14], Xu [15], and others, meanwhile, we consult the education experts in the school, the selection basis of indicators is established. It is believed that such indicators should be selected, which represent the students' basic learning ability, reflect the students' ability to analyze and solve problems, reflect the students' ability to effectively use time, and the students' recognition of learning value. Therefore, the four first-class indicators are established, which are the academic performance, practical ability, learning efficiency, and learning attitude.

Each primary indicator includes specific secondary indicators. The academic performance includes test score and exam score. The practical ability includes computer skills, academic capacity, and manipulative ability. The learning efficiency includes learning goals, learning plans, learning methods, and time management.

**Figure 3:**
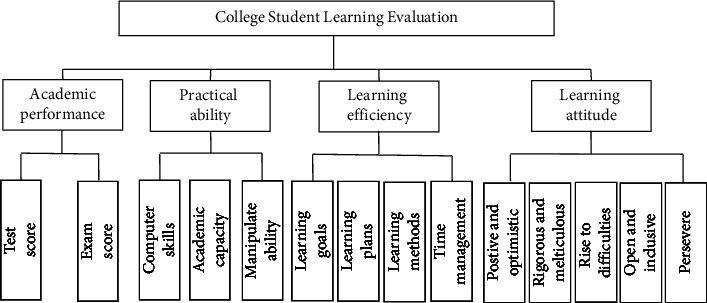
The learning evaluation index system of college students.

The learning attitude includes positive and optimistic, rigorous and meticulous, rise to difficulties, open and inclusive, and persevere. Therefore, a learning evaluation index system for college students is constructed, as shown in Figure 3:

Each secondary indicators can be divided into specific evaluation points, which are shown in Tables 3–6:

### 3.2. The Fuzzy Comprehensive Evaluation Set is Established

The learning evaluation results of college students are divided into four grades, they are perfect, very good, average, and bad, which is written as a judgment set: [16].

*V* = {perfect *V*_*1*_, very good *V*_*2*_, average *V*_*3*_, bad *V*_*4*_}.

### 3.3. The Weight of Each Index is Determined by Analytic Hierarchy Process

The weight of each indicator is different in college students learning evaluation, AHP was used to set the weight of each indicator: [16] The 1–9 scale method is used to compare and score the first-level indicators, the authors construct a pair comparison matrix of first order index *A*:

The weight vectors of each factor of the first-level index are obtained by sorting them in a single hierarchy. We use the root method to find the weight vector, set *W*′=[*a*_1_′, *a*_2_′, *a*_3_′,*a*_4_′], in which $a_{i}^{\prime }=\sqrt[{4}]{\prod_{j=1}^{4}a_{ij}}$ obtained *W*′=[0.562, 0.562, 2.659, 1.189] obtain *W*=[*a*_1_, *a*_2_, *a*_3_, *a*_4_]=[0.113, 0.113, 0.535, 0.239], through *W*′ normalization by *a*_*i*_=*a*_*i*_′/∑_*i*=1_^4^*a*_*i*_′ if the matrix *A* satisfies the consistency test, *W* is the weight vector of first–level index which are academic performance, practical ability, learning efficiency, and learning attitude. Compute whether the matrix *A* satisfies the consistency, results are as follows:

(9) $$\begin{array}{c} CR=0.002<0.1\text{.} \end{array}$$

The pairing comparison matrix *A* passes the consistency test. Similarly, the authors calculated the secondary indicators weights corresponding to the four primary indicators which is shown in Table 7. The total to pass the consistency test.

## 4. Experiment

This study evaluates the quality of college students. It is appropriate to select 6 to 10 evaluators. In this study, 10 evaluators are invited to form a learning evaluation group to evaluate the quality of students. The group includes 2 educational administrators, 4 professional teachers, and 4 students in the same class. The educational administrators should have the knowledge and ability of overall planning and implementation in talent training, curriculum construction, practical teaching, etc.; the teachers should have high attainments in professional fields with many years of teaching experience and certain teaching skills; the classmates should study in the same major as evaluation student. They get along with evaluation student day and night. Select a junior student of a major in our school. The evaluation group will evaluate and score according to his academic performance, practical ability, learning efficiency, and learning attitude. The specific scoring of the student is shown in Table 8.

Score the student's indicators on Table 8, four second-level evaluation matrices are obtained by the normalization method.

(10) $$\begin{array}{c} R_{1}=\left[\begin{array}{cccc} \begin{array}{c} 0\\ 0 \end{array} & \begin{array}{c} 0.9\\ 0.8 \end{array} & \begin{array}{c} 0.1\\ 0.2 \end{array} & \begin{array}{c} 0\\ 0 \end{array} \end{array}\right]\text{,}\\ R_{2}=\left[\begin{array}{cccc} \begin{array}{c} 0.6\\ 0\\ 0.4 \end{array} & \begin{array}{c} 0.3\\ 0.4\\ 0.4 \end{array} & \begin{array}{c} 0.1\\ 0.6\\ 0.2 \end{array} & \begin{array}{c} 0\\ 0\\ 0 \end{array} \end{array}\right]\text{,}\\ R_{3}=\left[\begin{array}{cccc} \begin{array}{c} 0\\ 0.3\\ 0.8\\ 0.1 \end{array} & \begin{array}{c} 0.4\\ 0.7\\ 0.2\\ 0.7 \end{array} & \begin{array}{c} 0.6\\ 0\\ 0\\ 0.2 \end{array} & \begin{array}{c} 0\\ 0\\ 0\\ 0 \end{array} \end{array}\right]\text{,}\\ R_{4}=\left[\begin{array}{cccc} \begin{array}{c} 0\\ 0.3\\ 0.1\\ 0.2\\ 0.1 \end{array} & \begin{array}{c} 0\\ 0.6\\ 0.9\\ 0.6\\ 0.6 \end{array} & \begin{array}{c} 1\\ 0.1\\ 0\\ 0.2\\ 0.3 \end{array} & \begin{array}{c} 0\\ 0\\ 0\\ 0\\ 0 \end{array} \end{array}\right]\text{.} \end{array}$$

The comprehensive evaluation result is obtained from *B*=*W*∘*R*, in which *W* is the weight vector of the secondary index. Calculate the comprehensive evaluation result of primary index *U*_1_: details are as follows:

(11) $$\begin{array}{c} B_{1}=W_{1}R_{1}=\left[\begin{array}{cc} 0.5 & 0.5 \end{array}\right]\left[\begin{array}{cccc} 0 & 0.9 & 0.1 & 0\\ 0 & 0.8 & 0.2 & 0 \end{array}\right]=\left[0,0.8500,0.1500,0\right]\text{.} \end{array}$$

Similarly, the comprehensive evaluation result of *U*_2_, *U*_3_, *U*_4_ are obtained.

(12) $$\begin{array}{c} B_{2}=W_{2}R_{2}=\left[0.3998,0.3429,0.2573,0\right]\text{,}\\ B_{3}=W_{3}R_{3}=\left[0.4836,0.3844,0.1320,0\right]\text{,}\\ B_{4}=W_{4}R_{4}=\left[0.1207,0.4203,0.4590,0\right]\text{.} \end{array}$$

The total evaluation matrix is obtained.

(13) $$\begin{array}{c} R=\left[\begin{array}{c} B_{1}\\ B_{2}\\ B_{3}\\ B_{4} \end{array}\right]=\left[\begin{array}{cccc} 0 & 0.8500 & 0.1500 & 0\\ 0.3998 & 0.3429 & 0.2573 & 0\\ 0.4836 & 0.3844 & 0.1320 & 0\\ 0.1207 & 0.4203 & 0.4590 & 0 \end{array}\right]\text{.} \end{array}$$

Finally, the comprehensive evaluation is made according to the weight of primary index *W*=[0.113, 0.113, 0.535, 0.239].

(14) $$\begin{array}{c} B=WR=\left[0.113,0.113,0.535,0.239\right]\left[\begin{array}{cccc} 0 & 0.8500 & 0.1500 & 0\\ 0.3998 & 0.3429 & 0.2573 & 0\\ 0.4836 & 0.3844 & 0.1320 & 0\\ 0.1207 & 0.4203 & 0.4590 & 0 \end{array}\right]=\left[0.3328,0.4409,0.2263,0\right]\text{.} \end{array}$$

It can be seen from the above results, the student had 44.09% “very good” evaluation, according to the principle of maximum membership degree, the student academic evaluation should be “very good.” The results is practical which has advantages over the simple analytic hierarchy process.

## 5. Conclusion

Learning centered evaluation directly focuses on the object of evaluation-students learning, because the students' learning and effectiveness are the final embodiment of teaching quality. [2] Teaching should pay more attention to students, and take promoting students' development and supporting students' learning as its fundamental starting point. We must firstly change the traditional definition of excellent teaching and establish the concept of teaching and evaluation that centered on students' learning in the process of promoting the reform of teaching evaluation of Colleges and universities in China [1]. The evaluation index system proposed in this paper is a general learning evaluation system for all students in colleges and universities, there must be an obvious difference for colleges and universities with different position, especially in their internal majors. Therefore, the index system set in this paper is not perfect. In the future, we should strengthen the research on more targeted unique evaluation indexes and calculation models.

The paper combines of analytic hierarchy process and fuzzy comprehensive evaluation method, which has certain advantages over only using the analytic hierarchy process [13] or only using the fuzzy comprehensive evaluation method [8]. However, the evaluation method used in this paper belongs to the category of fuzzy mathematics. The application flexibility and controllability of this evaluation method are restrained in view of theoretical system complexity, as well as the inevitable fuzziness and uncertainty of college students' learning evaluation. Therefore, developing and compiling the corresponding software operation package in line with the characteristics of College Students' learning evaluation system and simplifying the operation steps to the greatest extent will be the research focus of the combination of theory and practice in the future.

## Figures and Tables

**Table 1:** 1–9 scale methods.

Scale	Definition (compare factor *i* and *j*)
1	Factor *i* is as important as factor *j*
3	Factor *i* is slightly more important than factor *j*
5	Factor *i* is more important than factor *j*
7	Factor *i* is strong important than factor *j*
9	Factor *i* is absolutely important than factor *j*
2, 4, 6, 8	Between the above two situations
Reciprocal of the scale	The two factors are compared in reverse

**Table 2:** RI values of 3–9 order matrix.

Order number	RI
3	0.58
4	0.90
5	1.12
6	1.24
7	1.32
8	1.41
9	1.45

**Table 3:** The specific evaluation content of academic performance.

Primary index	Secondary indicators	Evaluation content
Academic performance	Test score	Including the scores of usual tests such as preclass tests, in class tests, and after-school assignments.
	Exam score	Generally refers to the final exam scores at the end of the course

**Table 4:** The specific evaluate content of academic performance.

Primary index	Secondary indicators	Evaluation content
Practical ability	Computer skills	Be familiar with basic computer operation skills and use computers to solve common problems in learning.
	Academic capacity	Be able to find scientific problems, summarize and refine literature content, and put forward their own views.
	Manipulative ability	Strong operation ability, able to integrate theory with practice.

**Table 5:** The specific evaluate content of learning efficiency.

Primary indicator	Secondary indicator	Evaluate content
Learning efficiency	Learning goals	Learning objective clear, thinking distinct, with stability and accessibility
	Learning plans	The study plan is scientific and fine, and the study life is reasonable and rich
	Learning methods	The study method is scientific and effective with outstanding personality and flexibility
	Time management	Scientific and strict time management, reasonable allocation, efficient use, with environmental adaptability

**Table 6:** The specific evaluate content of learning attitude.

Primary indicator	Secondary indicator	Evaluate content
Learning attitude	Positive and optimistic	Have a correct understanding of learning problems, have a positive attitude and be optimistic
	Rigorous and meticulous	The learning process is rigorous, scientific, meticulous, and strive for perfection
	Rise to difficulties	Dare to face problems directly, be not afraid of difficulties, and do not shrink back
	Open and inclusive	Look at problems from multiple perspectives, absorb everything and draw on the strengths of others
	Persevere	Persevere in learning goals, and the learning habits can continue uninterrupted

**Table 7:** The weight of each index and consistency test index.

Evaluation index	Weight of index	*λ*_max_	CI	RI	CR
*U*_1_–*U*_4_	(0.113, 0.113, 0.535, 0.239)	4.006	0.002	0.89	0.002
*u*_11_–*u*_12_	(0.500, 0.500)	2	0	0	0
*u*_21_–*u*_23_	(0.571, 0.286, 0.143)	3	0	0.58	0
*u*_31_–*u*_34_	(0.128, 0.128, 0.522, 0.114)	4.028	0.009	0.89	0.010
*u*_41_–*u*_45_	(0.348, 0.185, 0.097, 0.185, 0.185)	5.010	0.0025	1.12	0.0022

**Table 8:** Grade table of a junior student's learning situation.

Primary indicator	Secondary indicator	Evaluation set			
		Perfect	Very good	Average	Bad
*U*_1_	*u*_11_	0	9	1	0
	*u*_12_	0	8	2	0

*U*_2_	*u*_21_	6	3	1	0
	*u*_22_	0	4	6	0
	*u*_23_	4	4	2	0

*U*_3_	*u*_31_	0	4	6	0
	*u*_32_	3	7	0	0
	*u*_33_	8	2	0	0
	*u*_34_	1	7	2	0

*U*_4_	*u*_41_	0	0	10	0
	*u*_42_	3	6	1	0
	*u*_43_	1	9	0	0
	*u*_44_	2	6	2	0
	*u*_45_	1	6	3	0

## Data Availability

The data used to support the findings of this study are included within the article.
